# Supplementary material for: Transcriptional Regulation and Functional Characterization of the Plasmid-Borne oqxAB Genes in Salmonella Typhimurium
Source: Microbiol Spectr. 2022 Mar 22;10(2):e02170-21. doi: 10.1128/spectrum.02170-21 (PMC9045139; doi:10.1128/spectrum.02170-21)
Supplement: SUPPLEMENTAL FILE 1 — Supplemental material. Download SPECTRUM02170-21_Supp_1_seq12.pdf, PDF file, 0.6 MB [file spectrum02170-21_supp_1_seq12.pdf]

Supplementary materials

Transcriptional regulation and functional characterization of the plasmid-borne *oqxAB* genes in *Salmonella* Typhimurium

Marcus Ho-yin WONG[1]ǂ, Bill Kwan-wai CHAN[1,2]ǂ, Edward Wai-chi CHAN[1], Sheng CHEN[2*]

[1]State Key Laboratory of Chirosciences, Department of Applied Biology and Chemical Technology, The Hong Kong Polytechnic University, Hung Hom, Kowloon, Hong Kong.

[2]Department of Infectious Diseases and Public Health, Jockey Club College of Veterinary Medicine and Life Sciences, City University of Hong Kong, Kowloon, Hong Kong.

ǂContributed equally to the work.

Running Title: Regulation of plasmid-borne *oqxAB*

*Corresponding author: Sheng CHEN, Tel: (852)-3442-5782; Email: shechen@cityu.edu.hk

Keywords: *Salmonella*, OqxAB, PMQR, Regulation

Supplementary Table S1. Strains and plasmids used in this study.

Strain	Description
K. pneumoniae MGH78578	WT strain as template
S. Typhimurium ST06-53	Clinical isolate as template, *oqxAB*+
S. Typhimurium PY1	*S.* Typhimurium 14028s Type strain
PY1/pACYC-Duet	Vector control, Amp^R
PY1/paac+AB	*aac(6')-lb-cr*[EcoRI,HindIII] *and oqxAB*[NdeI,XhoI] cloned into pACYC, Amp^R
PY1/paac+ABRc	*aac(6')-lb-cr*[EcoRI,HindIII] *and oqxABR*c[NdeI,XhoI] cloned into pACYC, Amp^R
PY1/paac+ABRp	*aac(6')-lb-cr*[EcoRI,HindIII] *and oqxABR*p[NdeI,XhoI] from ST06-53 cloned into pACYC, Amp^R
PY1Δ*ramA*	*ramA*-deleted strain, Kan^R
PY1Δ*ramA*/pACYC-Duet	Vector control, Kan^R, Amp^R
PY1Δ*ramA*/paac+AB	*aac(6')-lb-cr*[EcoRI,HindIII] *and oqxAB*[NdeI,XhoI] cloned into pACYC , Kan^R, Amp^R
PY1Δ*ramA*/paac+pABRc	*aac(6')-lb-cr*[EcoRI,HindIII] *and oqxABR*c[NdeI,XhoI] cloned into pACYC , Kan^R, Amp^R
PY1Δ*ramA*/paac+pABRp	*aac(6')-lb-cr*[EcoRI,HindIII] *and oqxABR*p[NdeI,XhoI] from ST06-53 cloned into pACYC, Kan^R, Amp^R
PY1Δ*ramR*	*ramA*-deleted strain, Kan^R
PY1Δ*ramR*/pACYC-Duet	Vector control, Kan^R, Amp^R
PY1Δ*ramR*/paac+AB	*aac(6')-lb-cr*[EcoRI,HindIII] *and oqxAB*[NdeI,XhoI] cloned into pACYC , Kan^R, Amp^R
PY1Δ*ramR*/paac+pABRc	*aac(6')-lb-cr*[EcoRI,HindIII] *and oqxABR*c[NdeI,XhoI] cloned into pACYC , Kan^R, Amp^R
PY1Δ*ramR*/paac+pABRp	*aac(6')-lb-cr*[EcoRI,HindIII] *and oqxABR*p[NdeI,XhoI] from ST06-53 cloned into pACYC, Kan^R, Amp^R
PY1Δ*soxS*	*ramA*-deleted strain, Kan^R
PY1Δ*soxS*/pACYC-Duet	Vector control, Kan^R, Amp^R
PY1Δ*soxS*/paac+AB	*aac(6')-lb-cr*[EcoRI,HindIII] *and oqxAB*[NdeI,XhoI] cloned into pACYC , Kan^R, Amp^R
PY1Δ*soxS*/paac+pABRc	*aac(6')-lb-cr*[EcoRI,HindIII] *and oqxABR*c[NdeI,XhoI] cloned into pACYC , Kan^R, Amp^R
PY1Δ*soxS*/paac+pABRp	*aac(6')-lb-cr*[EcoRI,HindIII] *and oqxABR*p[NdeI,XhoI] from ST06-53 cloned into pACYC, Kan^R, Amp^R

Supplementary Table S2. Primers used in this study.

Primer	Sequence (5'-3')
Knockout	
ramA-KP1	GAGCCGCTGACGAGTTTGATAGAGGGGAGAGCACGATGACT<br>**GTGTAGGCTGGAGCTGCTTCG**
ramA-KP2	GTTGTTTTGTTTATGGTTTCTGTTGCTCGGCGCGCTGGAA<br>**TCCATATGAATATCCTCCTTAG**
soxRS-KP1	CGCGGCGTTCAGTATTGTCAGGGATGGCACTTTGCGAAG<br>**GTGTGTAGGCTGGAGCTGCTTCG**
soxRS-KP2	ATACAACCGTCCAGCTCATCGCGCAACGCCACCAGC<br>**TCCATATGAATATCCTCCTTAG**
Knockout Verification	
V-ramA-F	GCGATAAGCTGTCTCACAAT
V-ramA-R	TGCTGATGGCGTTGCTCTCC
V-soxRS-F	GCGGCTAAAAATCATTGC
V-soxRS-R	CAAACCGGAACCTCCACCAC
pKD4-k1	CAGTCATAGCCGAATAGCCT
pKD4-k2	CGGTGCCCTGAATGAACTGC
Cloning	
aac-F	CGATGAATTCGTACCGGAACAACGTGATTG
aac-R	CGATAAGCTTTTAGGCATCACTGCGTGTTC
oqxAB-ABRp-F	CGATCATATGGCAGCTCCATCAGCAAAAGG
oqxAB-ABRp-R	CGATCTCGAGAAAGGCTGCCTCATCGCTAA
oqxAB-ABRc-F	CGATCATATGGCGGCCGGTTAAAAGCATC
oqxAB-ABRc-R	CGATCTCGAGGGGGGTATGTCCCCTGTTC
oqxAB-AB-F	CGATCATATGGCAGCTCCATCAGCAAAAGG
oqxAB-AB-R	CGATCTCGAGCGGTTTTTTTGTTATCTGCTGCAGG
qRT-PCR	
rrsG-F	GTTACCCGCAGAAGAAGCAC
rrsG-R	CACATCCGACTTGACAGACC
oqxA-RT-F	GCATGAAAAAGGGCCAGGTGCT
oqxA-RT-R	CGCGGGAGACGAGGTTGGTATG
oqxB-RT-F	GCGGTGGTGCTGGTAGTGATCC
oqxB-RT-R	CGCCACCACGATACCGATAGCC
oqxR-RT-F	GCAGCGAACCCGAGCTTTATCC
oqxR-RT-R	CGCGGACGGCCAAGATGAATTG
5’RACE	
OqxR-GSP1	GCGAGCGAGGACGTTTAACG
OqxR-GSP2	AGCCTGCTCTGCTTCGTCGG
OqxR-GSP3	GAAGTACCAGCAGGCGTTGG
TSS Verification	
oqxR-TSSVeriF	CTGCGGTGCCAAAAAGAACAAGA
oqxR-TSSVeriR	CCGTCACGAGTTAGCGGAACC

Supplementary Table S3. ***oqxAB*** **positive** ***Salmonella*** **Typhimurium stool isolates from hospitalized patients.**

Isolates	Resistant gene		MIC (μg/mL)		
	aac(6')-Ib-cr	*oqxAB*	CIP	NA	OLA
Sal 06-53	+	+	2	≧128	≧512
Sal 06-57	+	+	0.1	32	≧512
Sal 07-37	+	+	2	≧128	≧512
Sal 07-38	+	+	2	≧128	≧512
Sal 07-43	-	+	0.025	16	256
Sal 07-45	+	+	0.25	≧128	128
Sal 07-47	+	+	2	≧128	≧512
Sal 07-57	+	+	2	≧128	≧512
Sal 08-10	+	+	1	≧128	≧512
Sal 08-11	+	+	0.1	32	≧512
Sal 08-14	+	+	2	≧128	≧512
Sal 08-15	+	+	2	≧128	≧512
Sal 08-19	+	+	1	≧128	≧512
Sal 08-27	+	+	2	≧128	256
Sal 08-48	+	+	2	≧128	≧512
Sal 08-52	+	+	2	≧128	≧512
Sal 08-53	+	+	2	≧128	256
Sal 08-54	+	+	1	≧128	128
Sal 08-58	-	+	0.5	≧128	≧512
Sal 09-37	+	+	2	≧128	≧512
Sal 09-39	+	+	2	≧128	≧512
Sal 09-46	+	+	4	≧128	≧512
Sal 09-49	+	+	2	≧128	≧512
Sal 09-50	+	+	2	≧128	≧512
Sal 09-52	+	+	2	≧128	≧512
Sal 09-53	+	+	2	≧128	256
Sal 09-58	+	+	2	≧128	≧512
Sal 09-59	+	+	2	≧128	≧512
Sal 10-3	+	+	2	≧128	≧512
Sal 10-8	+	+	2	≧128	256
Sal 10-9	-	+	0.25	≧128	128
Sal 10-10	-	+	2	≧128	≧512
Sal 10-11	+	+	2	≧128	≧512
Sal 10-13	+	+	0.5	≧128	128
Sal 10-14	+	+	2	≧128	≧512

Sal 10-29	-	+	0.5	≧128	≧512
Sal 10-63	+	+	1	≧128	≧512
Sal 10-75	-	+	1	≧128	≧512
Sal 11-3	+	+	2	≧128	256
Sal 11-58	+	+	4	≧128	512
Sal 11-117	+	+	2	≧128	512
Sal 11-120	+	+	2	≧128	256
Sal 11-131	+	+	4	≧128	512
Sal 11-135	-	+	2	≧128	256
Sal 11-136	+	+	4	≧128	512
Sal 12-12	-	+	0.5	≧128	512
Sal 12-14	+	+	4	≧128	512
ATCC25922	NA	NA	0.0078	4	8

Salmonella Typhimurium strains were isolated from the stool samples of hospitalized patients and the *oqxAB* positive strains were selected in this study. ATCC25922, the *E. coli* reference strain, was included as quality control in antimicrobial susceptibility tests. CIP, ciprofloxacin; NAL, nalidixic acid; OLA, olaquindox.
